# Deep learning and alternative learning strategies for retrospective real-world clinical data

**DOI:** 10.1038/s41746-019-0122-0

**Published:** 2019-05-30

**Authors:** David Chen, Sijia Liu, Paul Kingsbury, Sunghwan Sohn, Curtis B. Storlie, Elizabeth B. Habermann, James M. Naessens, David W. Larson, Hongfang Liu

**Affiliations:** 1 Division of Digital Health Sciences, Mayo Clinic, Rochester, MN USA; 2 Department of Health Science Research, Mayo Clinic, Rochester, MN USA; 3 Department of Colorectal Surgery, Mayo Clinic, Rochester, MN USA

**Keywords:** Translational research, Diagnosis, Prognosis, Signs and symptoms

## Abstract

In recent years, there is increasing enthusiasm in the healthcare research community for artificial intelligence to provide big data analytics and augment decision making. One of the prime reasons for this is the enormous impact of deep learning for utilization of complex healthcare big data. Although deep learning is a powerful analytic tool for the complex data contained in electronic health records (EHRs), there are also limitations which can make the choice of deep learning inferior in some healthcare applications. In this paper, we give a brief overview of the limitations of deep learning illustrated through case studies done over the years aiming to promote the consideration of alternative analytic strategies for healthcare.

## Introduction

Conventional machine learning (ML) has been applied to clinical decision support and medical discovery since the outset of the AI revolution.^1,2^ However, it is only in recent years with the advent of powerful computational tools, cheap electronic storage, and wide implementation of electronic health records (EHRs) that ML methods found themselves becoming accepted in mainstream medicine. Building upon conventional ML, “deep learning” methods promise another layer of the ability to automate difficult cognitive tasks, this time using “big data”.

One of the major limitations of conventional ML techniques is the requirement of sometimes complex processing (feature engineering) to extract the requisite discriminative features.^3^ Therefore, significant domain knowledge and data processing expertise were required to train non-deep learning models. Deep learning, however, is adept at learning abstract features directly from the raw data. Different layers of the network automatically learn abstract features representative of the data. A single well-designed and well-trained network can yield state-of-the-art results across many applications, without the need for significant domain knowledge.^4^ Many cognitive tasks previously thought to be limited to human operation due to the complexity of the data are now being automated by deep learning. Deep learning has made the prospect of self-driving vehicles feasible; beaten professionals in the game of Go, a board game with a huge scope of possible moves; achieved record accuracy in machine translation.^5–7^ It is no surprise that deep learning applications related to healthcare research has seen an explosive rise in the number of publications in the past several years.^8^ Correspondingly, there have been amazing accomplishments in the fields of medical image analysis, computational genomics, physiological signal analysis, medical data representation, and disease prediction due to the utilization of deep learing.^9–13^

It is clear that deep learning is an extremely powerful tool for learning complex, cognitive problems.^9,14^ However, it is not a comprehensive tool for all healthcare analytics applications. Several past commentaries on deep learning for clinical applications touch on how data issues such as low volume, high sparsity, and poor quality can limit the efficacy of deep learning methods.^15–19^ We concur with these ideas, and go a step further in focusing on the temporal aspect of healthcare data. We find that conventional ML tools can achieve comparable, if not better performance in this context despite the complex nature of the data. We present a wide range of representative use cases of ML solving clinical problems. We hope to demonstrate that although deep learning can be applied to many of these fairly standard problems, conventional ML methods may provide simpler, cheaper, and more useful method for data modeling.

### Clinical contexts

We review several limitations of deep learning tools illustrated with specific examples from prior work: prediction of postoperative bleeding following colorectal surgery (CRS-POB),^20^ prediction of childhood asthma diagnosis, remission, and reoccurance (A-DRR),^21^ prediction of time to first treatment for patients diagnosed with chronic lymphocytic leukemia (CLL-TFT),^22^ prediction of ICU mortality (ICU-M) using a publically available dataset,^21,23^ and finally prediction of opioid mis-use (Opioid).^24^ Each study was approved by Mayo Clinic’s Institutional Review Board. All patients in the CRS-POB, A-DRR, CLL-TFT, and Opioid datasets consented to use of their medical records for research purposes. The data used for these projects are summarized in Table 1. The data range from retrospective data extracted from Mayo Clinic Clinical Data Warehouse, a longitudinal regional clinical dataset, and a publically available dataset.^23,25^ Each dataset is complex and time-varying, making each problem ideal candidates for a data-driven ML approach. In each case we compare several traditional ML techniques such as logistic regression (LR), Bayesian network (BN), support vector machine (SVM), random forest (RF), and gradient boosting machine (GBM), with deep neural networks, including multi-layer perceptron (MLP) and long short term memory (LSTM), to predict clinically important events.

**Table 1:** Measures of dataset size, number of variables, and percentage of missing values

Dataset	# of cases	# of variables	Median # of time points	Median % Missing/variable		Max % Missing/variable	
				Time-varying	Tabular	Time-varying	Tabular
CRS-PSC	13399	117	71	21.3	99.9	100	49.6
A-DR*	4013	51	83	0	0	0	0
CLL-TFT	737	31	6	0	0	89.6	29.9
ICU-M	4000	41	24	21.6	24.0	95.7	94.9
Opioid^‡^	142377	836	32	0	0	0	0

Percentage of missing values are split between time-varying data and the condensed tabular data

*Features from clinical notes, no mentions considered negative result

^‡^No codes considered negative result

The methodology for modeling each clinical problem was fairly consistent across each project. A time-varying and corresponding single-time point dataset was created for each project. The single-time point dataset was created by taking maximum, minimum, mean, as well as maximum, minimum, and mean change in time for each time-varying variable. Following standard data cleaning, several ML algorithms were used to predict the outcomes of interest. The hyperparameters of each ML model were tuned using cross-validation. Specific to MLP models, we tuned the number of hidden layers, and varied the number of nodes in each hidden layer. For both MLP and LSTM models, we further tuned learning rate, drop out, activation function, loss function, and number of training epochs. The search grid can be found in the supplementary materials. The optimization metric for the MLP and LSTM models was accuracy. Areas under the receiver operating curve (AUROC) were used to assess the performance of the different models.

### Patient volume

Despite the enormous steps forward which deep learning has taken many cognitive tasks, deep learning has its own set of drawbacks. It is widely accepted that deep learning generally requires large volumes of data to accurately train the model.^26,27^ Applications for which deep learning has arguably provided the most benefit such as web search have access to large databases from which to learn from.^28^ Healthcare data however, is often highly limited in volume and quality due to sparsity of patient contact, variability in medical care, and privacy concerns.^29^

The need for data is reflected in the performance of the various models as shown in Table 2. In most cases, we found that conventional ML methods yielded better performance compared to the deep learning alternatives. We attribute this to the relatively small volume of training data. When the volume of training data is increased (such as in the CRS-PSC which has 3 times more data compared to other cases), deep learning methods become more competitive. Only in the large Opioid dataset (~ 100,000 cases) does deep learning alternative compare favorably to the conventional alternative, and even then cannot decisively outperform conventional methods. This is further demonstrated in Table 3, which shows comparison of model performance with different amounts of data on the CRS-PSC. Conventional ML methods also appear to have an upper limit in terms of accuracy whereas the LSTM model appears capable of further improving performance with increasing data.

**Table 2:** Predictive power of each model and their associated training time

App	Model	AUROC	Training time (s)
CRS-PSC	LR	0.735 ± 0.004	5.3
	BN	0.765 ± 0.004	48.6
	SVM	0.781 ± 0.003	33.1
	RF	0.812 ± 0.002	9.8
	GBM	**0.822** **±** **0.001**	13.8
	MLP	0.795 ± 0.003	219
	LSTM	0.805 ± 0.004	703
A-DRR	LR	0.875 ± 0.044	5.3
	SVM	0.884 ± 0.011	23.1
	RF	**0.947** ± **0.012**	10.8
	GBM	0.945 ± 0.011	23.8
	LSTM	0.845 ± 0.034	301.4
CLL-TFT	LR	0.795 ± 0.038	2.3
	SVM	0.843 ± 0.036	5.7
	RF	**0.924** **±** **0.004**	3.3
	GBM	0.817 ± 0.009	5.4
	LSTM	0.805 ± 0.017	46.5
ICU-M	LR	0.476 ± 0.074	4.3
	SVM	0.556 ± 0.051	12.7
	RF	0.618 ± 0.048	9.8
	GBM	**0.681** **±** **0.037**	16.4
	LSTM	0.646 ± 0.043	243.5
Opioid	LR	0.907 ± 0.002	NA
	SVM	0.904 ± 0.002	NA
	RF	0.875 ± 0.003	NA
	MLP	0.909 ± 0.002	NA
	LSTM	**0.909** **±** **0.002**	NA

Bolded values indicate best achieved metric for each project. Training times were not measured during the Opioid project

**Table 3:** Predictive power of each model based on percentage of data used to train

% of Data (N)	LR	BN	SVM	RF	GBM	MLP
10 (1340)	0.728	0.748	0.731	0.776	0.804	0.742
20 (2680)	0.727	0.749	0.758	0.797	0.811	0.762
40 (5360)	0.730	0.753	0.773	0.798	0.809	0.778
60 (8039)	0.732	0.759	0.772	0.801	0.812	0.786
80 (10,719)	0.731	0.764	0.779	0.808	0.818	0.791
100 (13,399)	0.735	0.765	0.781	0.812	0.822	0.795

### Patient variability

A large part of the appeal of EHRs is that the high variability of disease trajectories and patient care can be successfully captured and modeled. Despite the continual push to standardize patient care, there will always be a high level of variability between patients due to natural differences in disease presentation (e.g., location of cancer), variability in provider protocols (e.g., timing of laboratory tests), and even variability in desired outcomes (e.g., choice between attempting to treat terminal cancer or hospice care). All these sources of variability in disease progression greatly enlarges the search space which longitudinal models need to traverse. As seen in the CRS-PSC work, despite using a dataset which is fairly large for a healthcare related problem, the deep learning method could not achieve a better result compared to other ML methods.

The variability in patient data is further compounded by the use cases of clinical decision support tools. Due to the specialization of clinical practice, decision support models need to be limited in scope.^30^ For example, a general mortality prediction model built from all patients in multiple specialties would be difficult to evaluate by specific sub-populations, with no guarantee that the model would perform well on any specific sub-populations. However, limiting data from subspecialties may not yield enough data to train from, as exemplified in the CLL-TFT work.

Therefore, the vast majority of problems in healthcare which may benefit from ML contain significantly less data than appropriate for deep learning methodologies. The lack of relevant training data can be particularly true of smaller community institutions which do not have sufficient patient volume nor the resources to manually annotate data. One possible technique to address the lack of data is transfer learning.^31,32^ Transfer learning utilizes the architecture and weights of well-validated models as a starting point in training a new model for either a different task or different institution.^33^ Instead of building a model from scratch, and thereby requiring the acquisition and annotation of a completely new dataset, a smaller dataset can be used to efficiently update the weights in an existing model. This can greatly reduce the cost and effort required to build a dataset and retrain the model.^31^

### Data sparsity

In addition to the general lack of patient volume, many time-varying problems run into the issue of data sparsity. In the real world, providers often do not have a complete picture of a patient’s physiologic condition at any single time point, much less on a continuous basis. This tends not to be a problem for a human, as clinicians often can consciously or subconsciously impute patient status through other information. Harutyunyan et al. have argued that recurrent neural networks (RNN) can similarly utilize missing values.^34^ However, it is not clear that RNN models impute missing values in the same way that clinicians can. Che et al. directly incorporated a new parameter for missingness, therefore allowing the model to learn potential importance of the missing values.^35^

The other major strategy for dealing with missing values is imputation, but risks biasing the data (e.g., mean imputation) or is highly computational intensive (e.g., random forest model based). Che et al. shows the limitation of time-series imputation as certain methods such as cubic spline imputation may greatly reduce predictive accuracy.^35^

With low volumes of data, feature engineering used alongside conventional ML can provide a layer of denoising to improve information density and improve model performance. Although Wu et al. demonstrated that relative time between events can add predictive value for RNNs, we found that simple static classification can achieve better predictive results (as shown in Table 2).^21^ Asynchronous data collected over multiple hours can be compiled into a discrete measures, reducing the rate of missingness at each evaluated time point. Expert designed signal processing methods can also be utilized to identify previously known informative events, allowing for creation of highly specific data representations. Discretizing longitudinal data minimizes the number of parameters needed to model the data, therefore greatly reduce the amount of data needed for training, and greatly reducing the computational cost.

### Computational costs

Another significant disadvantage of deep learning is the associated data storage and computational infrastructure required to efficiently learn models. Longitudinal models such as RNNs have a large number of hyperparameters compared to even convolutional neural networks. This is further exacerbated by the sequential nature of RNNs and its inability to parallelize. Furthermore, as models become more complex to incorporate information such as relative time between events and data missingness, the need for data and computational power grows rather than shrinks. Parameters for relative time or missingness indicators represent a 1:1 increase in the number of parameters needed to be learned, both increasing the width of a model and also increasing the data required to sufficiently train the model. Newer models such as transformer may be more parameter and computationally efficient, but likely run into the same data complexity problems as conventional RNNs.^36^

Despite the large increases in computational capabilities and decreases in costs, it can still be financially oppressive to develop and maintain the computational infrastructure required to train deep models.

Most of this work (excluding the Opioid project) was completed on a single desktop equipped with a relatively inexpensive Intel Core i5-4570s CPU, and 8 GB of memory. The Opioid project was completed on a desktop equipped with an i5-4590S CPU and 16 GB of memory. As shown in Table 2, the deep learning method had the longest training time by far. Although we did not specifically benchmark the training time of the Opioid project, the deep learning models did take several hours to train on the CPU limited workstation. Although GPUs do greatly accelerate model development,^37^ GPUs enabled computing infrastructures are a significant expense, particularly for small healthcare organizations.

### Model interpretation

Healthcare models also require a degree of interpretability. Knowing the specific features driving a prediction can be important for clinical decision making and clear communication between patient and physician. Other ML methods can produce more interpretable models. For example, RF have specific measures of variable importance, allowing users to understand the relative contributions of variables to the overall prediction similar to the weights and p-values in LR. Although improving deep learning interpretability is an ongoing and prominent area of research, as of now deep learning models still tend to be black boxes.^38^

### Model evaluation and implementation

Another major criticism for research into applying ML in healthcare applications is that many techniques are not properly compared with clinical practice. Recently, several articles have shown comparable performance of certain medical tasks including prediction of all-cause mortality of patients admitted to intensive care unit, and diagnosis of pneumonia using chest X-rays.^14,39^ Despite the outsized claims often made, it is not yet evident that these advances in deep learning have (1) produced predictive performance similar to a human physician or (2) that deep learning is indisputably the ML method of choice. With respect to expert performance, Rajpurkar et al. compared a deep convolutional neural network against expert radiologist annotation of chest X-rays. The results showed that the model outperformed physicians in a blind read, where the radiologists were not given patient clinical background prior to reading the images. Although an impressive achievement, the direct applicability to practice is unclear as having patient history and other clinical data greatly increases diagnostic performance.

In addition to overselling the practicability of methods, the superiority of deep learning methods (or even a specific deep learning architecture) are often oversold as well. In the fore-mentioned case of automated diagnosis of pulmonary pathologies using chest X-rays, no comparison to other image learning architectures were made. Obviously, training of deep neural networks is computational expensive. However, this limits our understanding what is the state-of-the-art. In other cases such as predicting future mortality, baseline comparisons with competing models can be hidden or not well trained. The difference in model performance is typically small, so it is not at all apparent that using neural networks is worth the extra time and cost of training.

## Discussion

In taking these considerations into account, the choice of ML algorithm is highly important to achieve the most optimal (what is optimal can also vary between problems) results. It is sometimes easy to confuse the generalizability of deep learning methods for a catch-all data analytic technique. However, other ML methods can be much more computationally efficient, provide more interpretable models, and in the end prove to be more accurate. There is real clinical and scientific benefit to performing a thorough assessment of these conventional models, and should be included in academic publications.

Many of these problems originate from the disconnect which exists between data scientists and clinicians. Unlike purely cognitive applications such as driving or image search, healthcare is a patchwork of highly specialized processes and knowledge bases. Therefore, the desire to generate large, comprehensive models, using large comprehensive patient databases without fully understanding the final use case can lead to poor performance in practice. Unlike other industries were data scientists can work somewhat in isolation, data science in healthcare explicitly requires the cooperation of healthcare practitioners, informatics specialists, and data scientists. In-depth knowledge of current clinical workflow is needed such that models pull from relevant data sources, be trained on relevant patient cohort, and be applied at a relevant point during the clinical workflow.

We recognize that our perspectives are limited in several ways. For one, we included only a limited number of datasets. However, what we are advocating is not particularly bold, rather it is prudent to follow standard data science practice. Second, the deep learning models used here are not state-of-the-art and do not utilize methods such as bagging or boosting to improve performance. Although these methods can boost performance, the effect is generally marginal and does not significantly change our recommendations.^40–42^ Regardless of technique, comparison with established, popular techniques allow the ML and clinical communities properly assess the contribution of the methods.

## Conclusions

In conclusion, healthcare researchers should not be overly enthralled by the promises of deep learning. Although highly useful for certain tasks such as classifying medical images, deep learning is not suitable for all clinical data problems. In our experience across several clinical problems, conventional, off-the-shelf ML methods can be trained faster and have overall better performance when compared to deep neural networks. Unbridled excitement and confidence for deep learning can lead to unrealistic expectations, inappropriate applications, and ignorance of other more appropriate ML tools. Over confidence in deep learning without comparison with other methods can be detrimental to the progress of AI in clinical settings.

### Reporting summary

Further information on research design is available in the Nature Research Reporting Summary linked to this article.

## Supplementary information

Reporting Summary

Supplemental Table

## Data Availability

The CRS-PSC, A-DRR, CLL-TFT, and Opioid datasets utilized during this study are not publicly available due to privacy and security concerns. The data is not easily redistributable to researchers other than those engaged in the Institutional Review Board-approved research collaborations with Mayo Clinic. The ICU-M dataset is available for download at https://physionet.org/challenge/2012/.

## Supplementary information

**Supplementary information** accompanies the paper on the *npj Digital Medicine* website (10.1038/s41746-019-0122-0).

## Code availability

The code used for this study can be found at www.github.com/davidchenatmayo/ ForPubDM.
