# Supplementary material for: Deep learning and alternative learning strategies for retrospective real-world clinical data
Source: NPJ Digit Med. 2019 May 30;2:43. doi: 10.1038/s41746-019-0122-0 (PMC6550223; doi:10.1038/s41746-019-0122-0)
Supplement: Supplementary file 2 — Supplemental Table [file 41746_2019_122_MOESM2_ESM.docx]

| Dataset | Parameter | Search Space |
| --- | --- | --- |
| CRS-PSC | # Hidden layers | 1-3 |
|  | Learning rate | 0.00001-0.1 |
|  | Drop out | 0.3-0.7 |
|  | Activation function (MLP) | Sigmoid, ReLu |
|  | Optimizer | Adam, Adagrad, SGD |
|  | # Epochs | 250 |
| A-DR | # Hidden layers | 1-3 |
|  | Learning rate | 0.0001-0.1 |
|  | Drop out | 0.5 |
|  | Activation function (MLP) | Sigmoid, ReLu |
|  | Optimizer | Adam, SGD |
|  | # Epochs | 100 |
| CLL-TFT | # Hidden layers | 1-3 |
|  | Learning rate | 0.00001-0.1 |
|  | Drop out | 0.0-0.5 |
|  | Activation function (MLP) | Sigmoid, ReLu |
|  | Optimizer | Adam, Adagrad, SGD |
|  | # Epochs | 250 |
| ICU-M | # Hidden layers | 1-3 |
|  | Learning rate | 0.00001-0.1 |
|  | Drop out | 0.0-0.5 |
|  | Activation function (MLP) | Sigmoid, ReLu |
|  | Optimizer | Adam, SGD |
|  | # Epochs | 150 |
| Opioid | # Hidden layers | 1-3 |
|  | Learning rate | 0.00001-0.1 |
|  | Drop out | 0.5 |
|  | Activation function (MLP) | Sigmoid, ReLu |
|  | Optimizer | RMSprop |
|  | # Epochs | 250 |

Table 1 (Supplemental): Search space for hyper parameters of recurrent neural networks
